# Live imaging reveals the dynamics and regulation of mitochondrial nucleoids during the cell cycle in Fucci2-HeLa cells

**DOI:** 10.1038/s41598-017-10843-8

**Published:** 2017-09-12

**Authors:** Taeko Sasaki, Yoshikatsu Sato, Tetsuya Higashiyama, Narie Sasaki

**Affiliations:** 1 Division of Biological Science, Graduate School of Science, Nagoya University, Furo-cho, Chikusa-ku, Nagoya, Aichi 464-8602 Japan; 2 Institute of Transformative Bio-Molecules (WPI-ITbM), Nagoya University, Furo-cho, Chikusa-ku, Nagoya, Aichi 464-8601 Japan

## Abstract

Mitochondrial DNA (mtDNA) is organized in nucleoprotein complexes called mitochondrial nucleoids (mt-nucleoids), which are critical units of mtDNA replication and transmission. In humans, several hundreds of mt-nucleoids exist in a cell. However, how numerous mt-nucleoids are maintained during the cell cycle remains elusive, because cell cycle synchronization procedures affect mtDNA replication. Here, we analyzed regulation of the maintenance of mt-nucleoids in the cell cycle, using a fluorescent cell cycle indicator, Fucci2. Live imaging of mt-nucleoids with higher temporal resolution showed frequent attachment and detachment of mt-nucleoids throughout the cell cycle. TFAM, an mtDNA packaging protein, was involved in the regulation of this dynamic process, which was important for maintaining proper mt-nucleoid number. Both an increase in mt-nucleoid number and activation of mtDNA replication occurred during S phase. To increase mt-nucleoid number, mtDNA replication, but not nuclear DNA replication, was necessary. We propose that these dynamic and regulatory processes in the cell cycle maintain several hundred mt-nucleoids in proliferating cells.

## Introduction

Mitochondria are endosymbiotic organelles that possess their own DNA (mtDNA). The size of mtDNA has shrunk markedly over the course of evolution, although high copy numbers of mtDNA exist in individual cells. Such mtDNA should be precisely replicated and transmitted into the daughter cells through the cell cycle because mtDNA encodes essential subunits of the respiratory complex. Disorder of mtDNA maintenance causes mitochondrial dysfunction and leads to human diseases and aging^1^. In humans, there are thousands of copies of 16.6-kbp mtDNA in a cell and they are packaged by many proteins into hundreds of mt-nucleoids^2–5^. The mt-nucleoid is a unit of mtDNA transmission. Within dynamic mitochondrial networks, mt-nucleoids are semiregularly spaced, which is thought to be important for correct mtDNA transmission into the daughter cells at cell division^6–9^. A major mtDNA packaging protein, mitochondrial transcription factor A (TFAM), is a potential candidate for the regulation of mtDNA transmission^10, 11^. Knockdown of TFAM causes enlargement of mt-nucleoids and a decrease in their number, and also results in asymmetric transmission of mtDNAs into the two daughter cells^11^. In addition, the mt-nucleoid acts as a platform for mtDNA replication^12^. Some proteins related to mtDNA replication, such as DNA polymerase γ (POLG), mtDNA helicase Twinkle, and a single-stranded DNA-binding protein, mtSSB, are present in mt-nucleoids^13^. Recently, it has also been reported that such replication-related proteins accumulate at the mt-nucleoids with replicated mtDNAs, which are located at the endoplasmic reticulum (ER)–mitochondria contact site^12, 14^. However, there is little information about how the hundreds of mt-nucleoid are maintained during the cell cycle.

Cell cycle synchronization procedures are used to analyze the cell cycle. However, these procedures appear to affect mtDNA replication. Using synchronized cells, three different results have been reported; (1) mtDNA replication occurred constantly throughout the cell cycle^15, 16^, (2) mtDNA replication occurred throughout the cell cycle, but the activity peaks also exist at specific phases^17, 18^, (3) mtDNA replication occurred at specific phases^15^. Phases of the activity peak of mtDNA replication were different depending on cell-cycle-synchronization procedures^15, 18^. On the other hand, in unsynchronized cells, clear activity peaks were not observed^19^. Against this background of conflicting findings, the timing of mtDNA replication during the cell cycle has been discussed for more than 40 years.

Recently, a novel method for visualizing cell cycle stages was developed using a fluorescent cell cycle indicator, Fucci2^20, 21^. In this study, to investigate the maintenance of mt-nucleoids during the cell cycle without synchronization procedures, we used HeLa cells expressing Fucci2 (Fucci2 cells). We developed specific labeling of the mt-nucleoids with SYBR Green I in Fucci2 cells and the quantitative and highly sensitive detection of mtDNA replication using a thymidine analog, 5-ethynyl-2′-deoxyuridine (EdU). Using these imaging techniques, we revealed the dynamic behavior of mt-nucleoids for maintaining mt-nucleoid number properly and the coordination of regulation of mt-nucleoid number with mtDNA replication during the cell cycle.

## Results

### Low concentration of SYBR Green I selectively visualizes mtDNAs in the cell cycle

**Figure 1:**
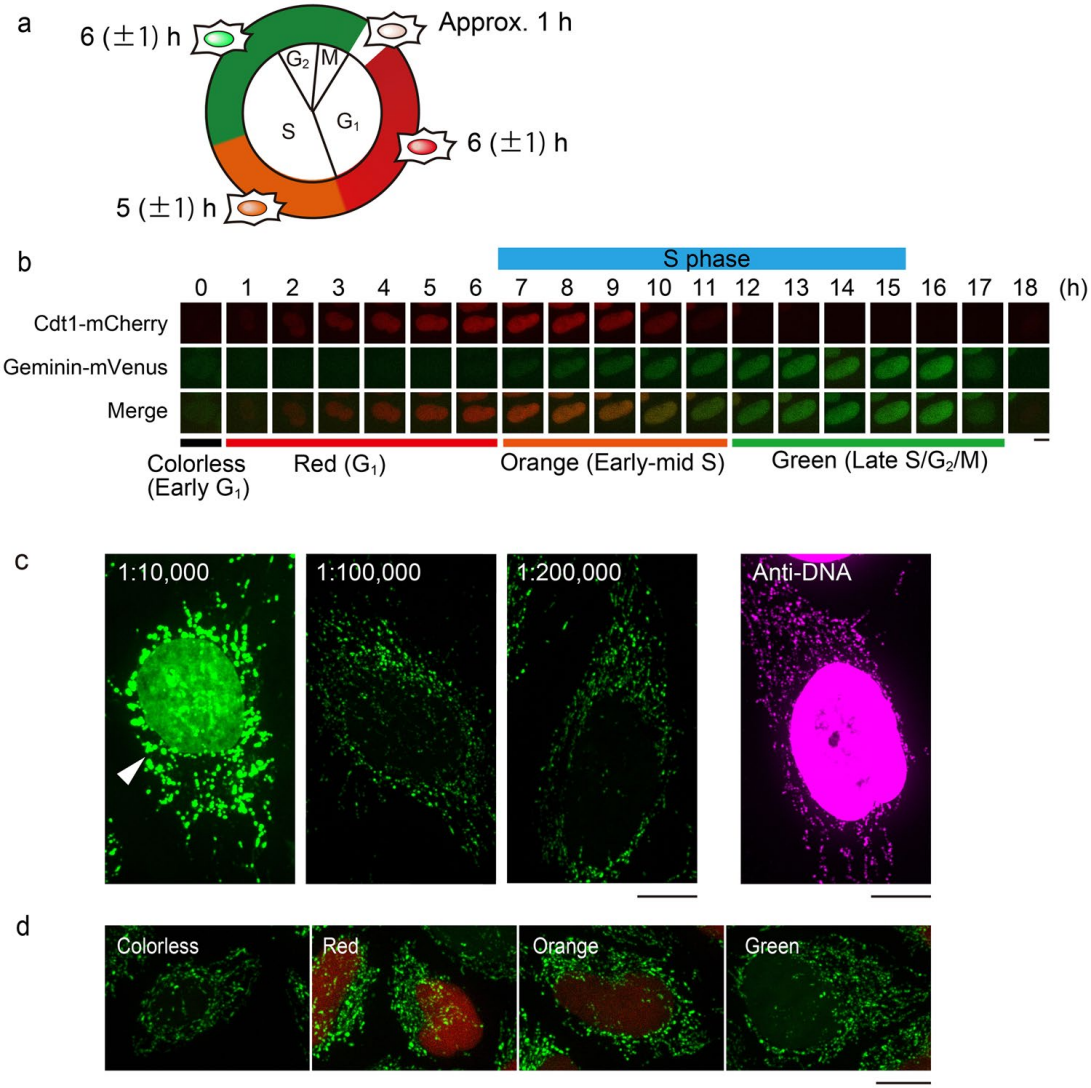
SYBR Green I can selectively visualize mt-nucleoids in Fucci2 cells. (**a**) Schematic representation of the Fucci2 cells used in this study. Average duration of each phase is shown. (**b**) Time-lapse series of typical fluorescent images during the cell cycle in Fucci2 cells. The duration of S phase was 9 h based on the experiment with EdU pulse labeling (blue bar, the detailed method is presented in the Materials and Methods section). Images showing the color of the nucleus are also presented as merged images. Scale bar, 10 μm. (**c**) Changes of staining pattern depending on the SYBR Green I concentration in normal HeLa cells. Dilutions of SYBR Green I are indicated above the images. DNAs were immunostained using anti-DNA antibodies in cells without staining of SYBR Green I (panel on the far right). Scale bars, 10 μm. (**d**) Selective visualization of mt-nucleoids in Fucci2 cells using a low concentration (1:300,000 dilution) of SYBR Green I. The color of the nucleus denoting the cell cycle phase is indicated in the upper left of each image. Scale bar, 10 μm.

Fucci2 cells were divided into four phases by the color of their nucleus. Colorless, red, orange, and green nuclei indicate early G_1_, G_1_, early-middle S, and late S/G_2_/M, respectively (Fig. 1a,b). Figure 1b shows a typical time course of the Fucci2 cells used in this study. The average duration of the cell cycle was 18 ± 2 h (n = 20 cells). Based on the nuclear color, the average duration of the early G_1_ phase (colorless) was 1 ± 0 h, G_1_ phase (red) was 6 ± 1 h, early-middle S phase (orange) was 5 ± 1 h, and late S/G_2_/M phase (green) was 6 ± 1 h (n = 10 cells).

To analyze mt-nucleoid dynamics during the cell cycle, we stained mt-nucleoids with SYBR Green I in Fucci2 cells. SYBR Green I has been used for visualizing living cells, such as those of animals, plants, and protists, and generally stains both nuclear DNA and mtDNA^22–25^. Such nuclear DNA staining hampers determination of the cell cycle phase in Fucci2 cells. However, we found that SYBR Green I selectively visualized mt-nucleoids at low concentration in living cells. When normal HeLa cells were stained at a 1:10,000 dilution of SYBR Green I, both nuclear DNA and mtDNA signals were observed. On the other hand, at 1:100,000 dilution or less, mt-nucleoids were selectively visualized without staining of the nucleus (Fig. 1c). To check influences of SYBR Green I staining on mt-nucleoid, normal size of mt-nucleoids were confirmed by immunostaining using anti-DNA antibodies. Compared with the immunostained mt-nucleoids, the size of mt-nucleoids stained with 1:10,000 dilution of SYBR Green I were abnormally enlarged, and the number of mt-nucleoids decreased. On the other hand, in the cells stained with 1:100,000 dilution or less of SYBR Green I, neither the enlargement of mt-nucleoids nor decrease in the number of mt-nucleoids were observed (Fig. 1c). By time-lapse imaging, we also found that mt-nucleoids enlarged at 1:100,000 dilution at 10 h, but not at 1:200,000 dilution (Supplementary Fig. S1). Next, we stained Fucci2 cells using SYBR Green I (Fig. 1d and Supplementary Fig. S1). Although the optimum concentration of SYBR Green I in Fucci2 cells was lower (1:300,000) than that in normal HeLa cells, we could observe both mt-nucleoids and the color of the nucleus of Fucci2 cells without any effects of their size and the number of mt-nucleoids (Fig. 1d).

### mt-nucleoids attach to and detach from each other with high frequency

**Figure 2:**
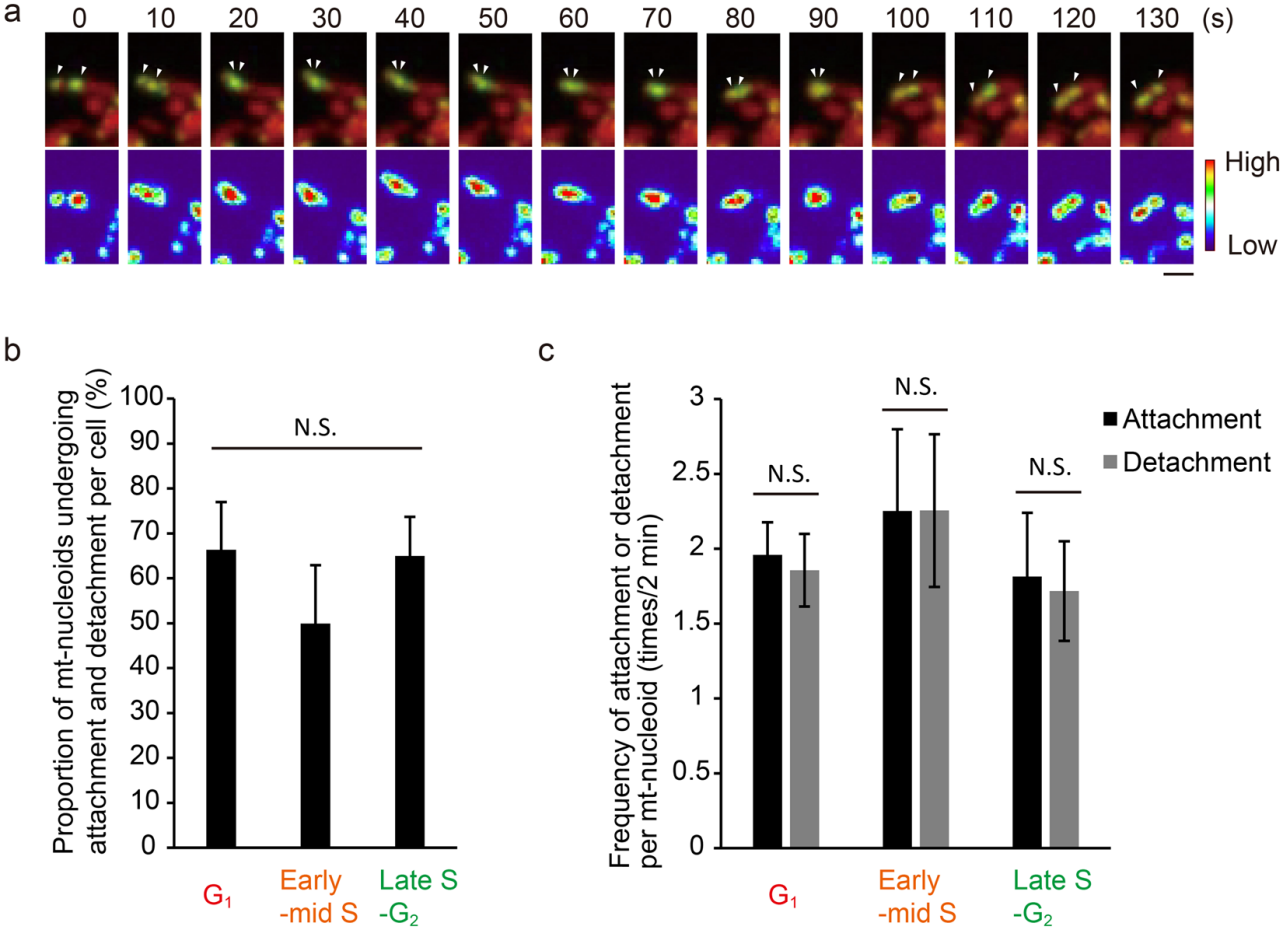
mt-nucleoids undergo frequent attachment and detachment during the cell cycle. (**a**) Time-lapse series of confocal images of mt-nucleoids (green) and mitochondria (red). mtDNAs were stained with SYBR Green I in HeLa cells expressing mitochondrially targeted DsRed (HeLa-Su9 cells). Lower panels show the fluorescence intensity of SYBR Green I as heat map images. Arrowheads show each mt-nucleoid. Scale bar, 1 μm. (**b**) Proportion of mt-nucleoids undergoing attachment or detachment per cell during the cell cycle mtDNAs were stained with SYBR Green I in Fucci2 cells. A total of 20 mt-nucleoids per cell were randomly selected and analyzed. Error bars indicate standard deviation (n_cells_ = 5 for each phase). The statistical significance of differences in attachment and detachment was examined by analysis of variance (p = 0.1005). N.S., not significant. (**c**) The frequency of attachment and detachment per mt-nucleoid for 2 min during the cell cycle. mtDNAs were stained with SYBR Green I in Fucci2 cells. mt-nucleoids undergoing attachment and detachment were analyzed for the number of attachments and detachments. A total of 20 mt-nucleoids per cell were randomly selected and analyzed. Error bars indicate standard deviation (n_cells_ = 5 for each phase). The statistical significance of differences in attachment and detachment was examined by paired t-test (G_1_, p = 0.1739; early-middle S, p = 0.9780; late S-G_2_, p = 0.2253). N.S., not significant.

To analyze the dynamics of mt-nucleoids, we performed time-lapse imaging of SYBR Green I-stained mt-nucleoids using spinning disk confocal microscopy (Fig. 2a and Video 1). Mt-nucleoids were actively moving within a mitochondrion. In addition, we often observed that mt-nucleoids in the same mitochondrion attached to each other, then moved together, and subsequently detached. Such attachment and detachment occurred independently of mitochondrial fission and fusion. To analyze the attachment and detachment of mt-nucleoids in detail, short-interval (<5 s) observation was performed (Fig. 2b,c). The duration of this experiment was limited to 2 min due to fading of the SYBR Green I. We observed that 50–66% of mt-nucleoids in a cell underwent attachment and detachment during the cell cycle in the 2-min observation period (Fig. 2b), although we could not analyze mt-nucleoids at M and early G_1_ phases due to the thickness of these cells. The frequencies of attachment and detachment were 1.8–2.3 times/mt-nucleoid and 1.7–2.3 times/mt-nucleoid in 2 min, respectively. In each cell cycle phase, there was no significant difference between the frequencies of attachment and detachment (Fig. 2c), suggesting that these processes occurred with equal frequency during the cell cycle.

**Figure 3:**
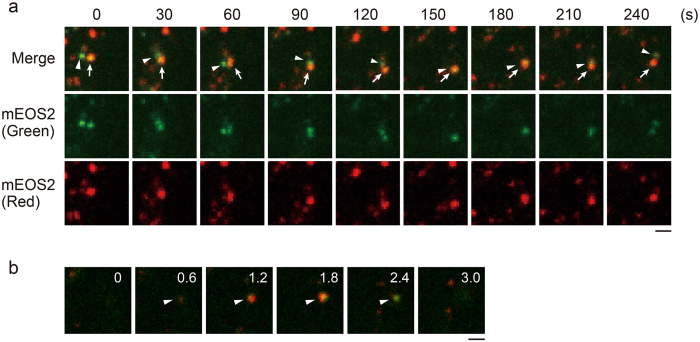
mt-nucleoids remain as discrete entities. (**a**) Time-lapse imaging of mt-nucleoids in TFAM-mEOS2 cells. Partial region of the cell was irradiated by a laser at 405 nm for photoconversion. Change of the colors of mt-nucleoids was not observed after attachment. Similar observations were obtained from 14 analyses. Arrowheads indicate non-photoconverted mt-nucleoids, and arrows indicate photoconverted ones. Scale bar, 1 μm. (**b**) Image sequence of the z stack shown in Fig. 3a at 180 s. The z stack position (μm) is shown in the upper right of each image. Scale bar, 1 μm.

Next, we examined whether the mt-nucleoids remain as discrete entities or mix their contents during attachment and detachment. We used an mt-nucleoid marker line expressing TFAM fused to mEOS2 (TFAM-mEOS2), allowing us to label mt-nucleoids by photoconversion. When a 405-nm laser was used to irradiate TFAM-mEOS2 cells, the fluorescence of mt-nucleoids was photoconverted from green to red (Supplementary Fig. S2). We found that overexpression of TFAM-mEOS2 induced mt-nucleoid enlargement and de novo TFAM-mEOS2 expression was detected after photoconversion (Supplementary Fig. S2). Therefore, the expression of TFAM-mEOS2 was controlled using the GeneSwitch system^26^, which is a mifepristone-inducible mammalian expression system. Using this system, the size of mt-nucleoids was found to be normal and de novo expression of TFAM-mEOS2 was not observed after photoconversion (Supplementary Fig. S2). When a 405-nm laser was irradiated to partial region of TFAM-mEOS2 cells, mt-nucleoids with a yellowish color were observed at the boundary region due to incomplete photoconversion. Next, we performed time-lapse imaging of the interaction of yellowish and green mt-nucleoids (Fig. 3). Figure 3a shows that yellowish and green mt-nucleoids underwent attachment at 150 s, moved together for more than 1 min, and then detached (Fig. 3a and Video 2). After this detachment, we found no apparent change of color of these two mt-nucleoids. Moreover, during the attachment, different colors were still observed in different z planes (Fig. 3b). These results suggest that mt-nucleoid proteins do not mix during the events of attachment and detachment and that mt-nucleoids remain as discrete entities during these processes.

**Figure 4:**
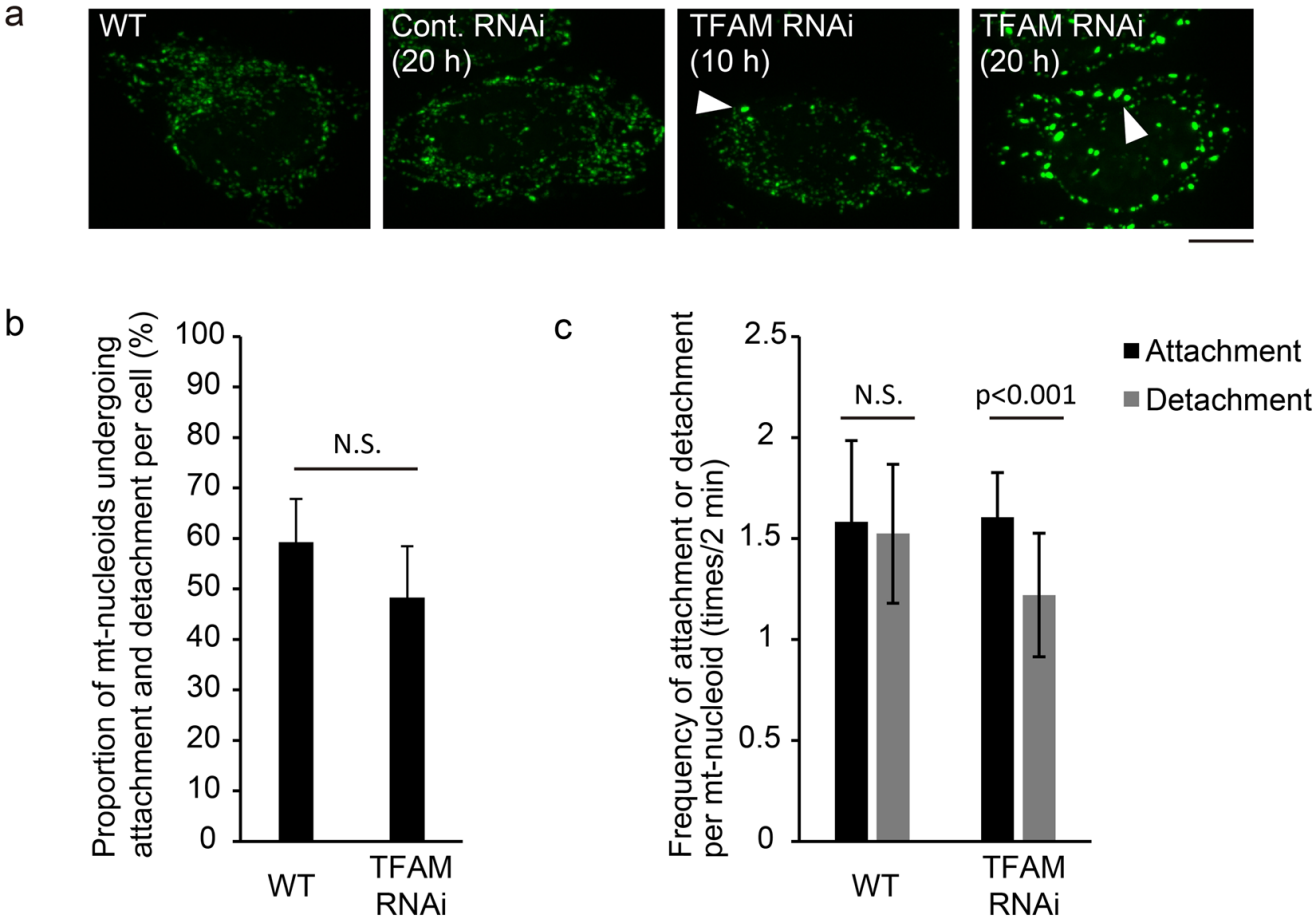
TFAM knockdown leads to reduced detachment of mt-nucleoids. (**a**) Effect of TFAM knockdown on mt-nucleoids. Knockdown of TFAM was performed in HeLa cells. mtDNAs were stained with SYBR Green I in TFAM-knockdown cells. Time after transfection of siRNAs is shown in the upper left of the images. Note that mt-nucleoid enlargement is observed from 10 h after transfection. Arrowheads indicate enlarged mt-nucleoids. Scale bar, 10 μm. (**b**) Proportion of mt-nucleoids undergoing attachment and detachment in wild-type and TFAM-knockdown HeLa cells. The significance of differences was examined by Student’s t-test (p = 0.0947). N.S., not significant. A total of 20 to 48 mt-nucleoids per cell were randomly selected and analyzed. Error bars indicate standard deviation (n_cells_ = 6 for each condition). (**c**) Frequency of attachment and detachment per mt-nucleoid in wild-type and TFAM-knockdown HeLa cells in 2 min. The frequency was analyzed in mt-nucleoids undergoing attachment and detachment. A total of 20 to 48 mt-nucleoids per cell were randomly selected and analyzed. Error bars indicate standard deviation (n_cells_ = 6 for each condition). The significance of differences in attachment and detachment was examined by paired t-test (WT, p = 0.1281; TFAM RNAi, p = 0.0007). N.S., not significant.

Our results imply that the remaining of mt-nucleoids as discrete entities is important for maintaining the number and size of mt-nucleoids unchanged by attachment and detachment. It has been reported that TFAM knockdown decreases the number of mt-nucleoids and increases their size^11^. Then, we next investigated whether there were abnormalities in the frequency of attachment and detachment of mt-nucleoids in TFAM-knockdown cells. When TFAM was knocked down in HeLa cells by transfection of TFAM siRNA, the low expression level of TFAM was observed at 10 h after siRNA transfection (Supplementary Fig. S4), and mt-nucleoids rapidly enlarged from 10 to 20 h after siRNA transfection (Fig. 4a). Therefore, we analyzed the movement of mt-nucleoids using TFAM-knockdown cells at 13 h after siRNA transfection. There was no apparent difference in the proportions of mt-nucleoids undergoing attachment and detachment between the wild-type and TFAM-knockdown cells (Fig. 4b). However, in the TFAM-knockdown cells, the frequency of detachment was significantly lower than that of attachment (Fig. 4c). This suggests that the ability of mt-nucleoids to undergo detachment was impaired in the TFAM-knockdown cells. Therefore, TFAM could be related to the regulation of attachment and detachment.

### The number of mt-nucleoids predominantly increase in the S phase

**Figure 5:**
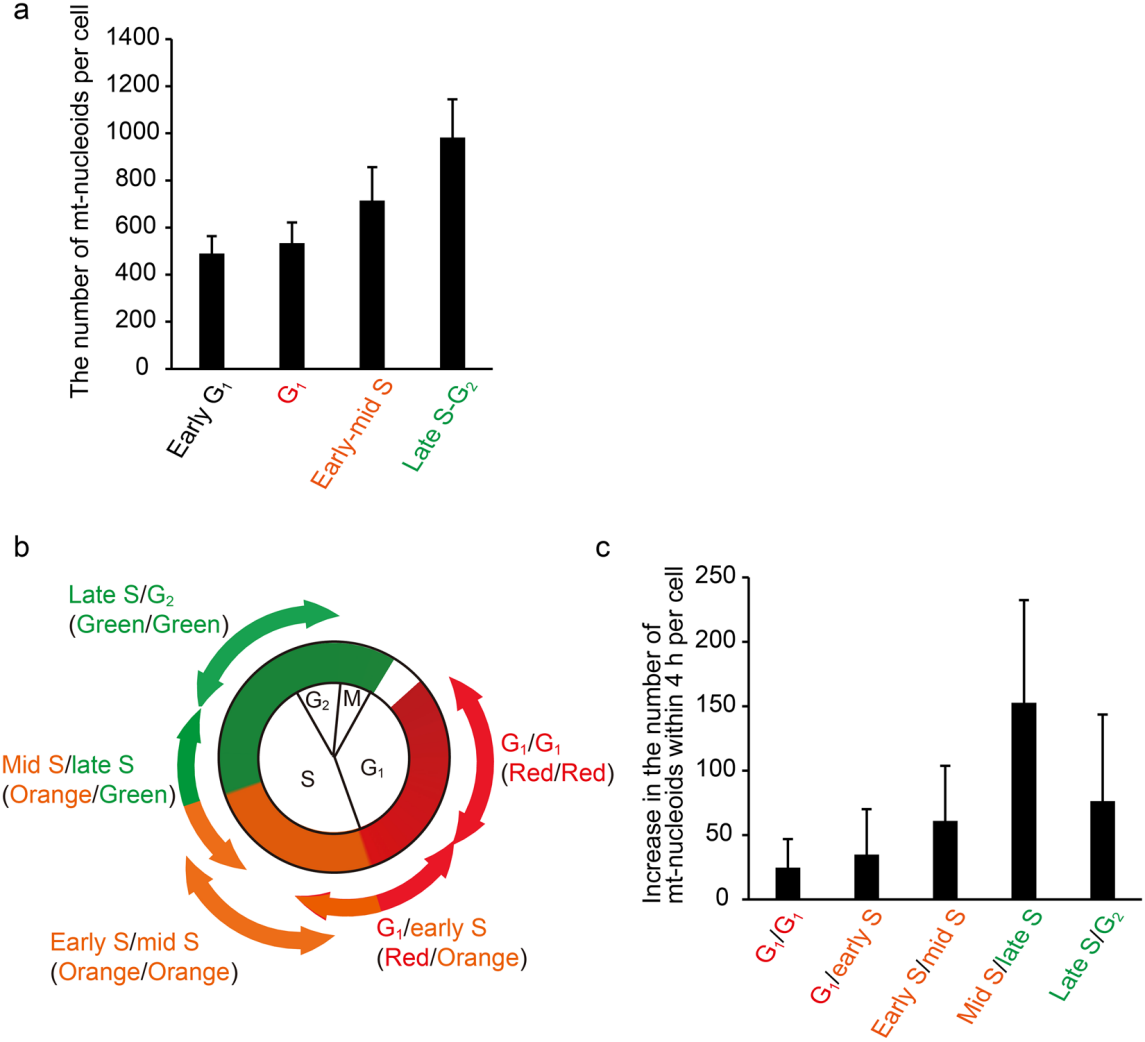
Predominant increase in the number of mt-nucleoids was observed during the S phase. (**a**) The number of mt-nucleoids per cell in each cell cycle phase. SYBR Green I-stained mt-nucleoids were counted in Fucci2 cells in each phase. Error bars indicate standard deviation (Early G_1_ n_cells_ = 19, G_1_ n_cells_ = 22, Early-middle S n_cells_ = 78, Late S-G_2_ n_cells_ = 42). (**b**) The period of time-lapse imaging for the experiment in Fig. 5c. Fucci2 cells were classified into five phases depending on the change in color of their nucleus. (**c**) Increase in the number of mt-nucleoids within 4 h during the cell cycle. We stained Fucci2 cells with 1:300,000 SYBR Green I, and performed time-lapse imaging at 4-h intervals in each cell cycle phase presented in Fig. 5b, after which we counted the mt-nucleoid number at 0 and 4 h. Error bars indicate standard deviation (n_cells_ = 10 for each phase).

To address the timing of the increase in the number of mt-nucleoids during the cell cycle, we counted the number of mt-nucleoids in Fucci2 cells stained with SYBR Green I at each cell cycle phase. The total numbers of mt-nucleoids in a cell were 490 ± 74, 534 ± 87, 714 ± 142, and 982 ± 163 in early G_1_, G_1_, early-middle S, and late S-G_2_, respectively (Fig. 5a). The number of mt-nucleoids at late S-G_2_ phase was almost double that in the G_1_ phase. Notably, a rather rapid increase in the number of mt-nucleoids was observed during the S phase compared with the G_1_ phase. To determine more details about the phase in which the number of mt-nucleoids increases, we also analyzed the changes of mt-nucleoid number in the same cell at 4-h intervals. In this experiment, cells could be classified into five phases depending on the change in color of their nucleus, as shown in Fig. 5b. The increases in number were 25 ± 22, 35 ± 35, 61 ± 43, 153 ± 80, and 77 ± 67 during the G_1_, G_1_ to early S, early S to middle S, middle S to late S, and late S to G_2_ phases, respectively (Fig. 5c). These results indicate that the number of mt-nucleoids predominantly increase during S phase, especially around the middle to late S phase.

### mtDNA replication occurs throughout the cell cycle with an activated peak in the S phase

To analyze when mtDNA replicates during the cell cycle, we visualized mtDNA replication in Fucci2 cells using EdU, which is incorporated into newly synthesized DNAs. In the conventional method for detecting incorporated EdU, a click reaction between the free alkynyl group on EdU and fluorescence-conjugated azide is used. To detect EdU signals by short pulse labelling, we enhanced the EdU signals by immunofluorescence staining using a rabbit primary antibody against fluorescence-conjugated azide and a secondary antibody against rabbit IgG, both of which were conjugated with Alexa Fluor 488. By using this procedure, the number and intensity of signals in the cytosol were increased compared with those by the conventional method (Supplementary Fig. S5). These signals were also detected by EdU treatment for only 10 min (Supplementary Fig. S6). These cytosolic signals were colocalized with mt-nucleoids labeled with anti-DNA, and not observed in the cells without EdU treatment (Supplementary Fig. S5), indicating that EdU signals in the cytosol were derived from newly synthesized mtDNA.

**Figure 6:**
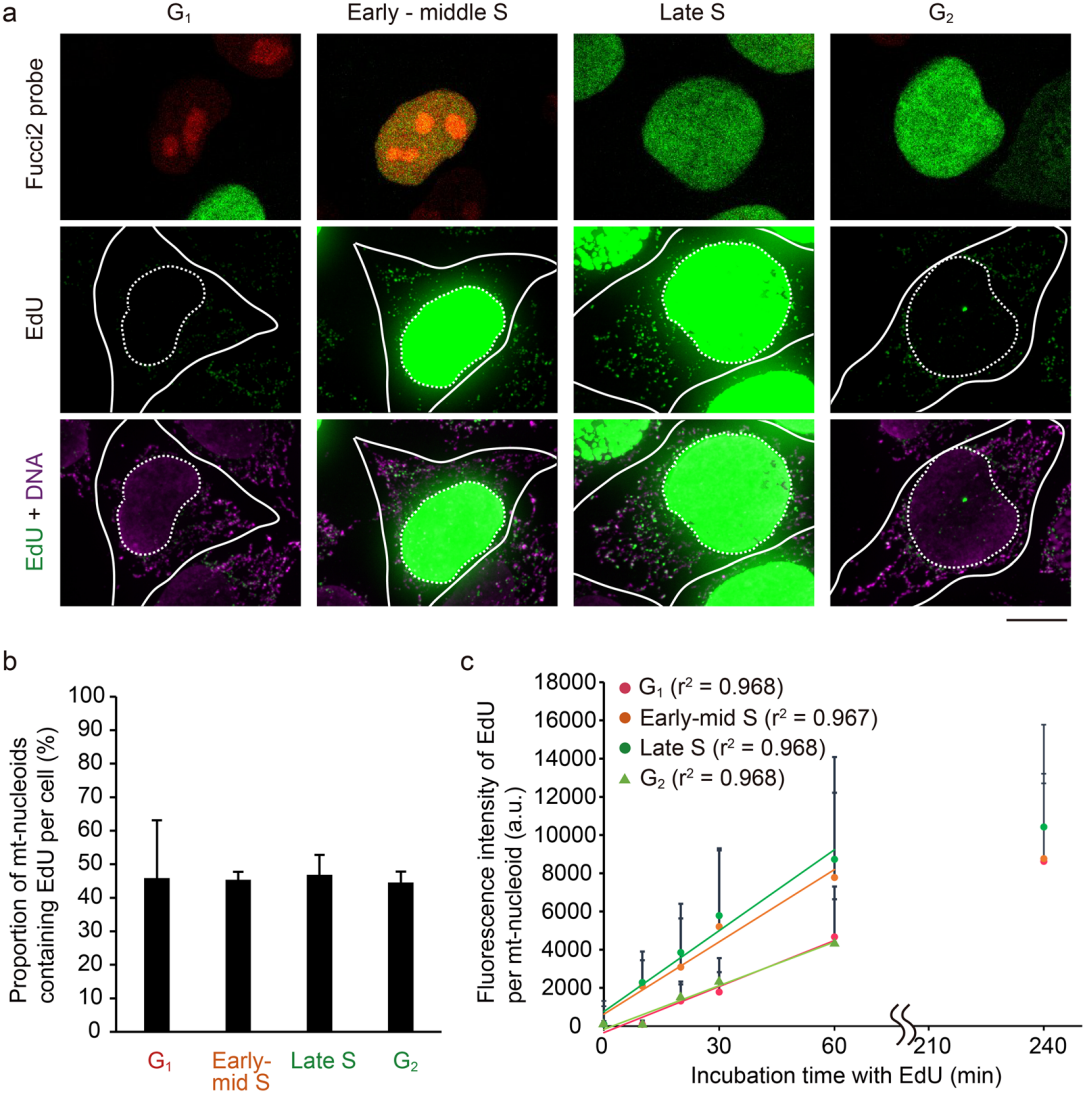
mtDNA replication occurs throughout the cell cycle, but the activity increases during the S phase. (**a**) Visualization of mtDNA replication in Fucci2 cells during the cell cycle. Fucci2 cells were incubated with 15 μM EdU for 60 min. After fixation, the color of the nucleus of Fucci2 cells was recorded, after which we performed signal amplification of EdU (green). We also performed immunostaining of DNA using anti-DNA antibodies (magenta), because signals of SYBR Green I in mt-nucleoids disappeared after fixation. Position of the nucleus (white dotted line) and cell shape (white line) are shown in each image. Scale bar, 10 μm. (**b**) Proportion of EdU-incorporating mt-nucleoids in a cell during the cell cycle. This proportion was calculated from the number of mt-nucleoids with EdU divided by the number of mt-nucleoids immunostained with anti-DNA antibodies in a cell. Error bars indicate standard deviation (G_1,_ n_cells_ = 7; early-middle S, n_cells_ = 9; late S, n_cells_ = 7; G_2_, n_cells_ = 7). (**c**) Fluorescence intensity of EdU signals in each mt-nucleoid during the cell cycle. The EdU intensity in each mt-nucleoid was analyzed using Fiji software. Approximately straight lines for 0–60 min of incubation are shown in the plot area, and the r^2^ value of each line is indicated in parentheses. The slopes of each approximation line are 80.31, 126.18, 141.26, and 67.53 for G_1_, early-middle S, late S, and G_2_, respectively. Error bars indicate standard deviation (n_mt-nucleoid_ = 200 from 10 cells for each plot).

Using this procedure, we analyzed the timing of mtDNA replication during the cell cycle in Fucci2 cells. In this experiment, we carried out immunostaining of EdU after recording the color of the nucleus in Fucci2 cells. We could distinguish between late S and G_2_ phases, which had the same fluorescent Fucci2 probe in the nucleus, by the EdU signals in the nucleus (Fig. 6a). When Fucci2 cells were incubated with EdU for 60 min, we detected EdU signals in mt-nucleoids throughout the cell cycle (Fig. 6a). The proportion of mt-nucleoids undergoing mtDNA replication was approximately 45% throughout the cell cycle (Fig. 6b). Then, we compared the relative amounts of EdU incorporation into mt-nucleoids during the cell cycle. When the incubation time of EdU was changed from 10 to 240 min, the intensities of EdU signals in mtDNAs increased linearly depending on the incubation time until 60 min, but upon prolonged incubation for 240 min, it was difficult to detect any further difference of signal intensity of EdU due to saturation (Fig. 6a,c, and Supplementary Fig. S6). At 60 min of pulse labeling with EdU, the intensities of EdU signals in the early-middle S and late S phase were approximately double those in the G_1_ and G_2_ phases (Fig. 6c). These results suggest that mtDNA replication occurs throughout the cell cycle, but its activity increases during the S phase.

### The total number of mt-nucleoids is regulated by mtDNA replication

**Figure 7:**
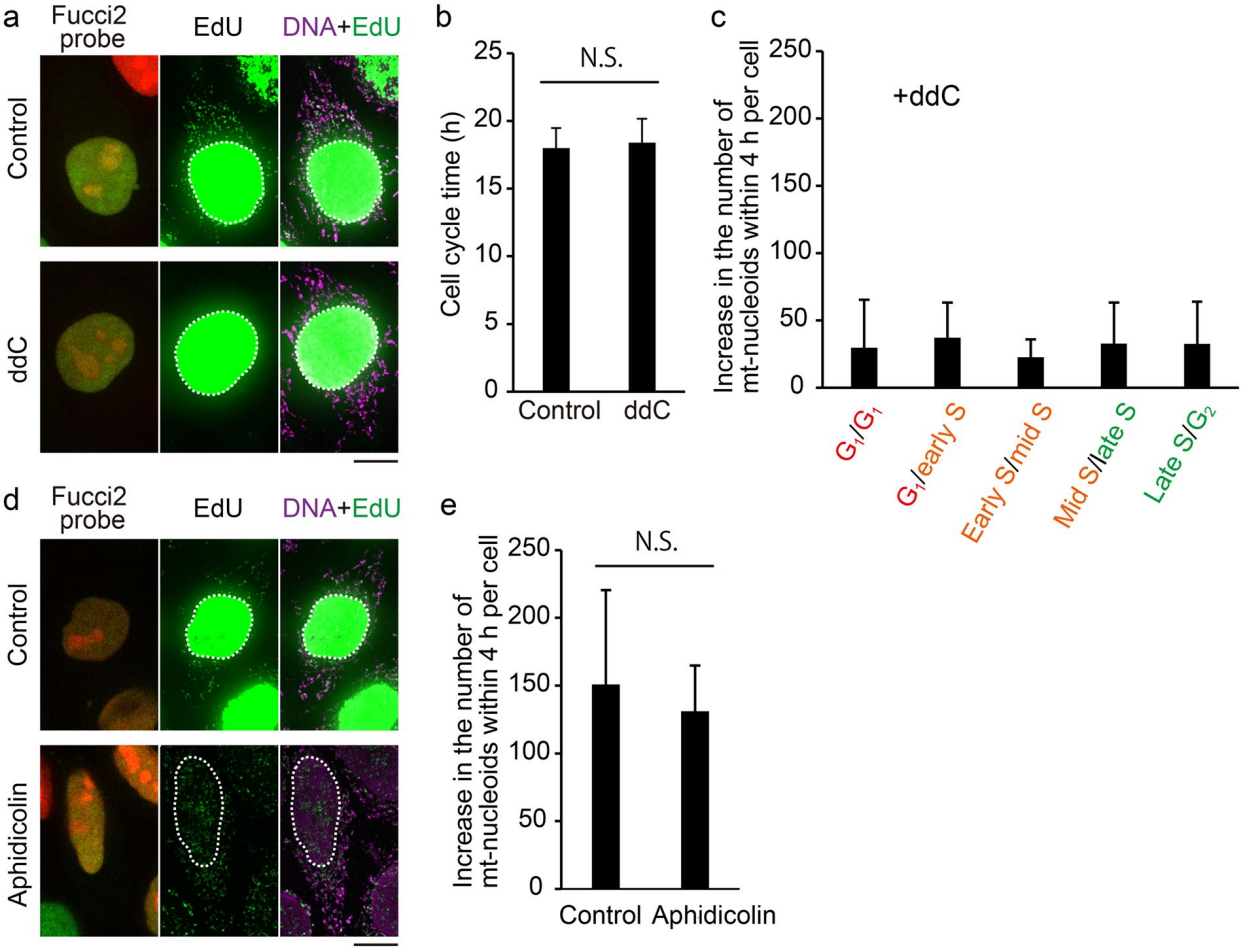
mtDNA replication is required for increase in the number of mt-nucleoids. (**a**) Effect of ddC treatment on mtDNA and nuclear DNA replication. Cells were treated with or without 100 μM ddC for 3 h, and subsequently incubated with 20 μM EdU for 1 h. Position of the nucleus (white dotted line) is shown in each image. Scale bar, 10 μm. (**b**) Effect of ddC treatment on cell cycle progression. Cells were treated with 100 μM ddC and time-lapse imaged at 1-h intervals. Error bars indicate standard deviation (n_cells_ = 20 for each phase). Statistical significance was examined by Student’s t-test. N.S., not significant (p = 0.4422). Scale bar, 10 μm. (**c**) Effect of ddC treatment on regulation of mt-nucleoid number. Fucci2 cells treated with ddC were time-lapse imaged at 4-h intervals. Error bars indicate standard deviation (n_cells_ = 10 for each bar). (**d**) Effect of aphidicolin treatment on mtDNA and nuclear DNA replication. Cells were treated with 15 μM aphidicolin (Aphidicolin) or 1:4000 DMSO (Control) for 10 h, and subsequently incubated with 20 μM EdU for 1 h. Position of the nucleus (white dotted line) is shown in each image. Scale bar, 10 μm. (**e**) Effect of aphidicolin treatment on regulation of mt-nucleoid number. The increase in the number of mt-nucleoids within 4 h was investigated during the phase in which the color of the nucleus changed from orange to green, in Fucci2 cells treated with 15 μM aphidicolin or 1:4000 DMSO for 10 h. Statistical significance was examined by Student’s t-test. N.S., not significant (p = 0.4325). Error bars indicate standard deviation (n_cells_ = 10 for each condition).

Thus far, we have shown that both the predominant increase in the number of mt-nucleoids and activation of mtDNA replication were observed in the S phase. To determine whether the increase in the number of mt-nucleoids is related to mtDNA replication, we also investigated the effect of a specific inhibitor of mtDNA replication, 2′,3′-dideoxycytidine (ddC), on the increase in the number of mt-nucleoids. We found that 3 h of treatment with 100 μM ddC completely suppressed mtDNA synthesis (Fig. 7a). Under these conditions, cell cycle progression was not affected, within at least the duration of one cell cycle (Fig. 7b). We performed time-lapse imaging at 4-h intervals to calculate the number of mt-nucleoids in the ddC-treated cells (Fig. 7c). The predominant increase in the number of mt-nucleoids in the S phase shown in untreated cells (Fig. 5d) disappeared in the ddC-treated cells.

In addition, we investigated the effect of a specific inhibitor of nuclear DNA polymerases α, δ, and ε, aphidicolin^27, 28^, on the increase in the number of mt-nucleoids. When cells were treated with 15 μM aphidicolin for 10 h, we observed severe reduction of EdU incorporation into the nucleus, but mtDNA replication was still observed in all treated cells (Fig. 7d). Under these conditions, the color of the nucleus was not frozen and progressed from orange to green, as previously reported^29^. Next, we performed time-lapse imaging to investigate the changes of mt-nucleoid number during the stage showing the predominant increase in the number of mt-nucleoids, in which the color of the nucleus changed from orange to green. The number of mt-nucleoids in the aphidicolin-treated cells increased similarly to that in the control cells within 4 h (Fig. 7e). This suggests that the blocking of nuclear DNA replication by aphidicolin does not affect either mtDNA replication or increase in the number of mt-nucleoids. Taking these findings together, we concluded that mtDNA replication, but not nuclear DNA replication, is indispensable for increase in the number of mt-nucleoids.

## Discussion

In this study, to analyze the regulation of maintenance of mt-nucleoids during the cell cycle, we developed new methods for the visualization of mt-nucleoids and mtDNA replication during the cell cycle without cell cycle synchronization using Fucci2 cells. Live imaging with higher temporal resolution using SYBR Green I showed that mt-nucleoids constantly attached to and detached from each other. The similar movement of mt-nucleoids by staining of mt-nucleoids using ethidium bromide (EtBr)^6^. However, it has been reported that EtBr staining induces enlargement of mt-nucleoids^30^. Such enlargement of mt-nucleoids was not observed at 1:200,000 dilution of SYBR Green I (Fig. 1c). Therefore, comparing with EtBr, SYBR Green I is suitable for live imaging of mt-nucleoids. Throughout the cell cycle, the attachment and detachment of mt-nucleoids occurred very frequently; about 60% of mt-nucleoids in a cell underwent attachment and detachment in 2 min (Fig. 2b, c). It is calculated that approximately 300–600 mt-nucleoids in a cell undergo attachment and detachment in 2 min, because there are about 500 (early G_1_) to 1000 (late S/G_2_) mt-nucleoids in a cell (Fig. 5a). During attachment and detachment, the mt-nucleoid protein, TFAM-mEOS2, didn’t mix (Fig. 3). This suggests that mt-nucleoids are maintained as discrete entities in spite of the frequent attachment and detachment.

The discreteness of mt-nucleoids in proliferating cells has also been shown by the fact that their mtDNAs do not mix^31^. Gilkerson *et al*.^31^ reported that, after the fusion of two homoplasmic cell lines carrying nonoverlapping partial deletions of mtDNAs, mtDNAs were not mixed between the mt-nucleoids, and mtDNA genotypes in a cell rapidly shifted to near homoplasmy within 32 days. This genetic autonomy of discrete mt-nucleoids provides a good explanation for the rapid genetic shift of mtDNA^32^. On the other hand, a previous report using a separate fluorescent protein showed that TFAM was mixed between mt-nucleoids after cell fusion in longer observations (6 h)^33^. We also observed that the green and red versions of TFAM-mEOS2 were completely mixed throughout an entire cell 4–6 h after photoconversion (Supplementary Fig. S3). It has been known that mitochondrial proteins can be mixed within mitochondrial networks, which undergo frequent fission and fusion^34^. TFAM is a member of the high-mobility group (HMG) proteins, which are highly mobile, and continuously move among chromatin by frequent dissociation and association^35, 36^. Therefore, it is possible that TFAM moved independently of mtDNAs and diffused in mitochondrial network like other mitochondrial proteins during the long-term observation.

The frequency of attachment and detachment was similar to each other (about 2 times/mt-nucleoid) throughout the cell cycle (Fig. 2c). However, in TFAM-knockdown cells, which exhibited decrease in the number of mt-nucleoids and increase in their size, the frequency of detachment was significantly lower than that of attachment (Fig. 4c). This suggests that the regulation of attachment and detachment should be important to maintain the proper number and size of mt-nucleoids. It has been reported that abnormalities in the size and number of mt-nucleoids by TFAM-knockdown lead to unequal partitioning into two daughter cells^11^. Therefore, the regulation of attachment and detachment might be also important for precise mtDNA transmission in proliferating cells.

It is possible that TFAM is involved in the mechanism for the attachment and detachment of mt-nucleoids through the regulation of detachment. Since one mitochondrion contains several mt-nucleoids and mt-nucleoids are actively moving, mt-nucleoids are likely to accidentally attach with each other. Therefore, the regulation of the detachment of attached mt-nucleoids by TFAM might be important for keeping mt-nucleoids discrete. TFAM is known as a major DNA packaging protein, which binds to mtDNA every 10~20 bp^4, 37^. This suggests that the organization of mtDNA and the composition of proteins in mt-nucleoids are related to the regulation of detachment.

Using live imaging of Fucci2 cells stained with SYBR Green I, we showed the regulation of the number of mt-nucleoids during the cell cycle. The total number of mt-nucleoids doubled from the G_1_ to the G_2_ phase, and a rather rapid increase was observed from the S to the G_2_ phase (Fig. 5a). Time-lapse imaging of a single cell showed that the peak of increase in the number of mt-nucleoid existed in the middle to late S phase. These results suggested that mt-nucleoid segregation was activated around the middle-late S phase. In this phase, it is calculated that the frequency of mt-nucleoids segregation was no more than 1 times per minute in a cell on average, because the number of mt-nucleoids increased by about 150 within 4 h (Fig. 5c). This frequency is much lower than that of attachment and detachment (300-600 times in a cell per minute); thus, it might be difficult to detect mt-nucleoid segregation in our system. To detect mt-nucleoid segregation, establishing a new method would be needed to arrow us to track mt-nucleoids for a long time.

We also successfully determined the timing of mtDNA replication by developing an enhanced method of EdU staining. Another thymidine analog, bromodeoxyuridine (BrdU), has long been used for visualizing mtDNA replication, but the severe DNA denaturation step associated with BrdU detection makes difficult to perform reproducibly, resulting in low quantitativity. On the other hand, the EdU procedure, which is not accompanied by this denaturation step, is suited to quantitative analysis. However, only faint EdU signals were detected in mt-nucleoids by the conventional EdU detection method due to the lack of sensitivity (Supplementary Fig. S5). Therefore, to define the timing of mtDNA replication in detail, we established the short pulse labeling of EdU to perform quantitative analysis of mtDNA replication (Fig. 6c). Using this new technique, we first showed that the activity of mtDNA replication approximately doubled during the S phase compared with that in the G_1_ phase in HeLa cells without cell cycle synchronization. This result suggests the mechanism for connection of the cell cycle progression to mtDNA replication. Recently, mtSSB, which is required for mtDNA replication, was shown to interact with cyclin-dependent kinase (CDK) subunit proteins, CKS1 and CKS2, and to be phosphorylated by CDK^38^. CDKs and cyclins are known as the main regulators of cell cycle progression, and mitochondrial size, activity, morphology, and protein import have been reported to be regulated by CDKs and cyclins^39–41^. It is possible that these factors connect mtDNA replication with the cell cycle.

Both activation of mtDNA replication and increase in the number of mt-nucleoids were observed at the S phase in HeLa cells. In addition, mtDNA replication, but not nuclear DNA replication, is required for increase in the number of mt-nucleoids (Fig. 7). These results suggest the mechanism of the coordination of regulation of mt-nucleoid number with mtDNA replication. It is known that some proteins necessary for mtDNA replication, such as POLG, POLGβ, mtSSB and Twinkle, are localized to mt-nucleoids^42^. Knockdown of these proteins induce the mtDNA depletion^42–46^. In addition, knockdown of POLGβ, which is an accessory subunit of DNA polymerase γ and enhances polymerase processivity, caused an increase in the number of mt-nucleoids, while overexpression of this protein induced a decrease in the number^46^. This suggests that POLGβ might act as a negative regulator of the number of mt-nucleoids. It is possible that the negative regulation by POLGβ could be canceled by mtDNA replication. Further study will be needed to elucidate the molecular mechanism of this regulation.

## Methods

### Cell culture

HeLa and HeLa.S-Fucci2 cells (RCB0007 and RCB2867, respectively; RIKEN Cell Bank, Tsukuba, Japan) were cultured in Dulbecco’s Modified Eagle Medium (D-MEM; Wako, Osaka, Japan) containing 10% fetal bovine serum (FBS). The HeLa cell line expressing mitochondrial-targeted DsRed^40^ was cultured in D-MEM containing 10% FBS and 1 μg/ml puromycin. TFAM-mEOS2 cells were cultured in D-MEM containing 10% FBS, 100 μg/ml hygromycin B, and 100 μg/ml zeocin. These cell lines were maintained at 37 °C in 5% CO_2._

### Microscopy settings and image acquisition

All fluorescent images in this study were obtained using a spinning disk confocal system (CellVoyager CV1000; Yokogawa Electric, Tokyo, Japan) equipped with 488-nm and 561-nm diode lasers. Confocal images were acquired with a 100× oil immersion objective lens (UPLSAPO 100XO, WD = 0.13 mm, NA = 1.40; Olympus, Tokyo, Japan) or a 100× silicone immersion objective lens (UPLSAPO100XS, WD = 0.20 mm, NA = 1.35; Olympus). Exposure time was 100 ms and fluorescence was acquired through band-pass filters, BP525/50 for mVenus, Alexa488, OregonGreen, or pre-photoconverted mEOS2 and BP617/73 for mCherry, Alexa594, or post-photoconverted mEOS2. Images were processed with CV1000 software (Yokogawa Electric) to create maximum-intensity projection images. The brightness and contrast of all images were adjusted by Fiji (http://fiji.sc/).

### Determination of cell cycle phase in Fucci2 cells

Fucci2 cells were plated onto an eight-well cover glass chamber (AGC Techno Glass Co., LTD., Japan﻿,﻿ 2 × 10^4^ cells per well) and cultured at 37 °C. After 1 day, time-lapse imaging of the cells was performed at 1 h intervals. Nuclear color of the nucleus in Fucci2 cells were analyzed using Fiji software. To determine the duration of the S phase in Fucci2 cells, cells were incubated with EdU for 10 min, and click reaction and signal amplification of EdU were performed. Next, we determined the proportion of cells in the S phase (51%, n_cells_ = 251). We calculated the duration of the S phase (approximately 9 h) by multiplying the total cell cycle time (18 h) by the proportion of cells in the S phase.

### Analysis of the dynamics and the number of mt-nucleoids using SYBR Green I staining

Cells were plated onto an eight-well cover glass chamber (2 × 10^4^ cells per well) and cultured at 37 °C for 24 to 36 h. SYBR Green I (Thermo Fisher Scientific, Japan) was diluted 1:10 times with dimethyl sulfoxide, and subsequently diluted with D-MEM (SYBR Green I solution) 1:30,000 times for HeLa.S-Fucci2 or 1:20,000 times for HeLa-Su9 cells. Cells were incubated with SYBR Green I solution at 37 °C for 5 min in a CO_2_ incubator and washed three times with fresh growth medium. After SYBR Green I staining, cells were incubated at 37 °C in a CO_2_ incubator of CV1000 for 3 h and then analyzed.

For analysis of the frequencies of attachment and detachment of mt-nucleoids, images of SYBR Green I-stained cells were acquired every 5 s using 10 z-plane sectioning with 0.6-μm intervals. The maximum-intensity projection images were uploaded into Imaris (Bitplane AG, Zurich, Switzerland) and tracking of mt-nucleoids was performed manually. A total of 20 to 48 mt-nucleoids per cell were randomly selected and analyzed.

For analysis of the number of mt-nucleoids, fluorescent images of SYBR Green I-stained cells were acquired using 35 or 61 z-plane sectioning with 0.3-μm intervals. The number of mt-nucleoids per cell was counted manually on maximum-intensity projection images.

### Labeling of mt-nucleoids by photoconversion using TFAM-mEOS2 cell line

To make TFAM-mEOS2 inducible, we used the GeneSwitch system (Thermo Fisher Scientific). The subcloned *mEOS2* fragment was inserted into multicloning sequences of pGeneV5/His between NotI and AgeI sites. A human *TFAM* fragment was inserted into pGene-mEOS2 using BamHI and NotI sites. The *mEOS2* fragment was amplified by PCR from pRSETa mEOS2 (Plasmid #20341; Addgene, Cambridge, MA, U.S.A.) using the primers 5′-AACTGCGGCCGCATGAGTGCGATTAAGCCAG-3′ and 5′-GCAGACCGGTTATCGTCTGGCATTGTC-3′. The human *TFAM* fragment was amplified by PCR from human cDNA using the primers 5′-GGGATCCCACCATGGCGTTTCTCCGAAG-3′ and 5′-TAGATGCGGCCGCCACACTCCTCAGCACCAT-3′. The pGene-TFAM-mEOS2 and commercially provided pSwitch vectors were cotransfected into HeLa cells by jetPEI (Polyplus Transfection, NY, USA), in accordance with the manufacturer’s protocol. Cells were cloned in D-MEM containing 10% FBS, 100 μg/ml hygromycin B, and 100 μg/ml zeocin using the serial dilution method. The expression of TFAM-mEOS2 was checked by observation using CV1000 in the presence of 10 nM mifepristone; a clone that expressed TFAM-mEOS2 was chosen for the experiments as a TFAM-mEOS2 cell line.

For the experiments of photoconversion, TFAM-mEOS2 cells were plated onto an eight-well cover glass chamber (2 × 10^4^ cells per well). After 1 day, cells were incubated with 10 nM mifepristone for 23 h and then washed three times with fresh growth medium. After 2 h, photoconversion was performed by irradiation with a laser at 405 nm for 7 s using CV1000. For time-lapse imaging, the images were obtained every 30 s or 2 h using 10 z-plane sectioning with 0.6-μm intervals.

### Down-regulation of TFAM using RNAi

HeLa-Su9 cells were plated onto an eight-well cover glass chamber (1 × 10^4^ cells per well) and cultured for 1 day. Next, siRNAs (final conc. 20 nM) were transfected by Lipofectamine RNAi MAX (Thermo Fisher Scientific) into the cells, in accordance with the manufacturer’s protocol. The siRNAs were purchased from RNAi Inc. (Japan). For TFAM RNAi, the target sequence was 5′-AAGTTGTCCAAAGAAACCTGT-3′. For negative control siRNA targeting the luciferase gene, the target sequence was 5′-CGUACGCGGAAUACUUCGA-3′.

### Visualization of mtDNA replication using EdU

Fucci2 cells were plated onto an eight-well cover glass chamber (2 × 10^4^ cells per well) and cultured at 37 °C for 24 to 36 h. These cells were incubated in D-MEM with or without 15 to 20 μM EdU (Thermo Fisher Scientific) for 10 to 240 min.

To detect incorporated EdU into DNA, cells were fixed with 8% (wt/vol) paraformaldehyde in PBS (137 mM NaCl, 3 mM KCl, 8 mM Na_2_HPO_4_, 1.5 mM KH_2_PO_4_, pH 7) at 37 °C for 3 min, and permeabilized with 0.5% (vol/vol) Triton X-100 in PBS at room temperature for 10 min. After fixation and permeabilization, fluorescent images of the cells were obtained using CV1000 to record the colors of the nucleus of cells. EdU was detected using Click-iT Cell Reaction Buffer Kit (Thermo Fisher Scientific), in accordance with the manufacturer’s protocol. In brief, Click-iT reaction cocktail was created by mixing Click-iT cell reaction buffer, CuSO_4_, Oregon Green 488 azide (Thermo Fisher Scientific), and Click-iT cell reaction buffer additive, and this mixture was then added to the cells and incubated for 50 min at room temperature under dark conditions. The reaction cocktail was then removed, after which the cells were washed three times in PBS and incubated with rabbit Alexa Fluor 488-conjugated antibodies against Oregon Green (A-11090; Thermo Fisher Scientific) and mouse antibodies against DNA (MAB030; Merck Millipore, Billerica, MA, USA) in PBS with 1% (wt/vol) skimmed milk powder. The primary antibodies were detected with secondary antibodies (goat Alexa 488-conjugated anti-rabbit: A-11008, goat Alexa 594-conjugated anti-mouse: A-11032; Thermo Fisher Scientific). The fluorescent images of immunostained cells were acquired using 35 z-plane sectioning with 0.3-μm intervals. The number of fluorescent spots derived from mtDNAs was analyzed on maximum-intensity projection images. Since it was difficult to analyze mt-nucleoids that were located underneath or above the nucleus, we excluded these from the calculation of the proportion of EdU-incorporating mt-nucleoids.

For determination of the intensity of fluorescent spots derived from mtDNAs, 20 spots per cell were randomly selected and the mean intensity of each spot was measured using Fiji software. To avoid the effect of saturation of nuclear EdU signals, the background in the immediate vicinity of the EdU spot was analyzed and subtracted from the original intensity.

### Treatment of ddC and aphidicolin

Fucci2 cells were plated onto an eight-well cover glass chamber (2 × 10^4^ cells per well) and cultured at 37 °C for 24 h. These cells were incubated in D-MEM with 100 μM ddC (Wako) for 3 h or with 15 μM aphidicolin (Wako) for 10 h, and then EdU labeling and time-lapse imaging were performed. For EdU labeling, ddC- or aphidicolin treated cells were incubated with 20 μM EdU for 1 h. EdU detection was performed as described above. For time-lapse imaging, cells were stained with SYBR Green I three hours before imaging, and fluorescent images of SYBR Green I-stained cells were acquired at 4-h intervals using 35 z-plane sectioning with 0.3-μm intervals. The number of mt-nucleoids per cell was counted manually on maximum-intensity projection images.

### Statistics

In general, two-tailed Student’s *t-tests* were used. One-way ANOVA was used to compare the difference among three groups. For the analysis of frequency of attachment and detachment, two-tailed paired *t-test* was used. The data were considered statistically significant for p < 0.05.

### Data Availability

The data that support the findings of this study are available within the paper and its Supplementary Information files, or are available from the corresponding author upon reasonable request.

## Electronic supplementary material

Supplementary Information

Video 1

Video 2

## References

[1] Wallace DC (2010). Mitochondrial DNA mutations in disease and aging. Environ. Mol. Mutagen..

[2] Satoh M, Kuroiwa T (1991). Organization of multiple nucleoids and DNA molecules in mitochondria of human cell. Exp. Cell Res..

[3] Legros F, Malka F, Frachon P, Lombès A, Rojo M (2004). Organization and dynamics of human mitochondrial DNA. J. Cell. Sci..

[4] Kukat C (2011). Super-resolution microscopy reveals that mammalian mitochondrial nucleoids have a uniform size and frequently contain a single copy of mtDNA. Proc. Natl. Acad. Sci. USA.

[5] Bogenhagen DF (2012). Mitochondrial DNA nucleoid structure. Biochim. Biophys. Acta.

[6] Iborra FJ, Kimura H, Cook PR (2004). The functional organization of mitochondrial genomes in human cells. BMC Biology.

[7] Tauber J (2013). Distribution of mitochondrial nucleoids upon mitochondrial network fragmentation and network reintegration in HEPG2 cells. Int. J. Biochem. Cell Biol..

[8] Osman C, Noriega TR, Okreglak V, Fung JC, Walter P (2015). Integrity of the yeast mitochondrial genome, but not its distribution and inheritance, relies on mitochondrial fission and fusion. Proc. Natl. Acad. Sci. USA.

[9] Jajoo R (2016). Accurate concentration control of mitochondria and nucleoids. Science.

[10] Alam TI (2003). Human mitochondrial DNA is packaged with TFAM. Nucleic Acids Res..

[11] Kasashima K, Sumitani M, Endo H (2011). Human mitochondrial transcription factor A is required for the segregation of mitochondrial DNA in cultured cells. Exp. Cell Res..

[12] Rajala N, Gerhold JM, Martinsson P, Klymov A, Spelbrink JN (2014). Replication factors transiently associate with mtDNA at the mitochondrial inner membrane to facilitate replication. Nucleic Acids Res..

[13] Bogenhagen DF, Rousseau D, Burke S (2008). The Layered Structure of Human Mitochondrial DNA Nucleoids. J. Biol. Chem..

[14] Lewis SC, Uchiyama LF, Nunnari J (2016). ER-mitochondria contacts couple mtDNA synthesis with mitochondrial division in human cells. Science.

[15] Pica-Mattoccia L, Attardi G (1972). Expression of the mitochondrial genome in HeLa cells. IX. Replication of mitochondrial DNA in relationship to cell cycle in HeLa cells. J. Mol. Biol..

[16] Antes A (2010). Differential regulation of full-length genome and a single-stranded 7S DNA along the cell cycle in human mitochondria. Nucleic Acids Res..

[17] Lee S, Kim S, Sun X, Lee J-H, Cho H (2007). Cell cycle-dependent mitochondrial biogenesis and dynamics in mammalian cells. Biochem. Biophys. Res. Commun..

[18] Chatre L, Ricchetti M (2013). Prevalent coordination of mitochondrial DNA transcription and initiation of replication with the cell cycle. Nucleic Acids Res..

[19] Bogenhagen D, Clayton DA (1977). Mouse L cell mitochondrial DNA molecules are selected randomly for replication throughout the cell cycle. Cell.

[20] Sakaue-Sawano A (2008). Visualizing spatiotemporal dynamics of multicellular cell-cycle progression. Cell.

[21] Sakaue-Sawano A, Kobayashi T, Ohtawa K, Miyawaki A (2011). Drug-induced cell cycle modulation leading to cell-cycle arrest, nuclear mis-segregation, or endoreplication. BMC Cell Biol..

[22] Arimura S, Yamamoto J, Aida GP, Nakazono M, Tsutsumi N (2004). Frequent fusion and fission of plant mitochondria with unequal nucleoid distribution. Proc. Natl. Acad. Sci. USA.

[23] Nishimura Y (2006). Active digestion of sperm mitochondrial DNA in single living sperm revealed by optical tweezers. Proc. Natl. Acad. Sci. USA.

[24] Maeda-Sano K (2009). Visualization of Mitochondrial and Apicoplast Nucleoids in the Human Malaria Parasite Plasmodium falciparum by SYBR Green I and PicoGreen Staining. Cytologia.

[25] Ozawa S, Sasaki N (2009). Visualization of mitochondrial nucleoids in living human cells using SYBR Green I. Cytologia: international journal of cytology.

[26] Brown TA (2011). Superresolution fluorescence imaging of mitochondrial nucleoids reveals their spatial range, limits, and membrane interaction. Mol. Cell. Biol..

[27] Ikegami S (1978). Aphidicolin prevents mitotic cell division by interfering with the activity of DNA polymerase-alpha. Nature.

[28] Wright GE, Hübscher U, Khan NN, Focher F, Verri A (1994). Inhibitor analysis of calf thymus DNA polymerases alpha, delta and epsilon. FEBS Lett..

[29] Marcus JM, Burke RT, DeSisto JA, Landesman Y, Orth JD (2015). Longitudinal tracking of single live cancer cells to understand cell cycle effects of the nuclear export inhibitor, selinexor. Sci Rep.

[30] Ashley N, Poulton J (2009). Anticancer DNA intercalators cause p53-dependent mitochondrial DNA nucleoid re-modelling. Oncogene.

[31] Gilkerson RW, Schon EA, Hernandez E, Davidson MM (2008). Mitochondrial nucleoids maintain genetic autonomy but allow for functional complementation. J. Cell Biol..

[32] Cao L (2007). The mitochondrial bottleneck occurs without reduction of mtDNA content in female mouse germ cells. Nat Genet.

[33] Kasashima K, Endo H (2015). Interaction of human mitochondrial transcription factor A in mitochondria: its involvement in the dynamics of mitochondrial DNA nucleoids. Genes Cells.

[34] Ishihara N, Jofuku A, Eura Y, Mihara K (2003). Regulation of mitochondrial morphology by membrane potential, and DRP1-dependent division and FZO1-dependent fusion reaction in mammalian cells. Biochem. Biophys. Res. Commun..

[35] Parisi MA, Clayton DA (1991). Similarity of human mitochondrial transcription factor 1 to high mobility group proteins. Science.

[36] Catez F (2004). Network of dynamic interactions between histone H1 and high-mobility-group proteins in chromatin. Mol. Cell. Biol..

[37] Takamatsu C (2002). Regulation of mitochondrial D‐loops by transcription factor A and single‐stranded DNA‐binding protein. EMBO reports.

[38] Radulovic M, Crane E, Crawford M, Godovac-Zimmermann J, Yu VPCC (2010). CKS proteins protect mitochondrial genome integrity by interacting with mitochondrial single-stranded DNA-binding protein. Mol. Cell Proteomics.

[39] Wang C (2006). Cyclin D1 repression of nuclear respiratory factor 1 integrates nuclear DNA synthesis and mitochondrial function. Proc. Natl. Acad. Sci. USA.

[40] Taguchi N, Ishihara N, Jofuku A, Oka T, Mihara K (2007). Mitotic phosphorylation of dynamin-related GTPase Drp1 participates in mitochondrial fission. J. Biol. Chem..

[41] Harbauer AB (2014). Mitochondria. Cell cycle-dependent regulation of mitochondrial preprotein translocase. Science.

[42] Young MJ, Copeland WC (2016). Human mitochondrial DNA replication machinery and disease. Current Opinion in Genetics & Development.

[43] Tyynismaa H (2004). Twinkle helicase is essential for mtDNA maintenance and regulates mtDNA copy number. Hum Mol Genet.

[44] Humphrey DM (2012). Alternative oxidase rescues mitochondria-mediated dopaminergic cell loss in Drosophila. Hum. Mol. Genet..

[45] Ruhanen H (2010). Mitochondrial single-stranded DNA binding protein is required for maintenance of mitochondrial DNA and 7S DNA but is not required for mitochondrial nucleoid organisation. Biochim. Biophys. Acta.

[46] Di RM (2009). The accessory subunit of mitochondrial DNA polymerase gamma determines the DNA content of mitochondrial nucleoids in human cultured cells. Nucleic Acids Res..

