# Supplementary material for: Live imaging reveals the dynamics and regulation of mitochondrial nucleoids during the cell cycle in Fucci2-HeLa cells
Source: Sci Rep. 2017 Sep 12;7:11257. doi: 10.1038/s41598-017-10843-8 (PMC5595809; doi:10.1038/s41598-017-10843-8)
Supplement: Supplementary file 1 — Supplementary Information [file 41598_2017_10843_MOESM1_ESM.pdf]

Supplementary Information

Live imaging reveals the dynamics and regulation of mitochondrial nucleoids during the cell cycle in Fucci2-HeLa cells

Taeko Sasaki[1], Yoshikatsu Sato[2], Tetsuya Higashiyama[1,2], Narie Sasaki[1]

[1]Division of Biological Science, Graduate School of Science, Nagoya University, Furo-cho, Chikusa-ku, Nagoya, Aichi 464-8602, Japan

[2]Institute of Transformative Bio-Molecules (WPI-ITbM), Nagoya University, Furo-cho, Chikusa-ku, Nagoya, Aichi 464-8602, Japan

Supplementary Figures

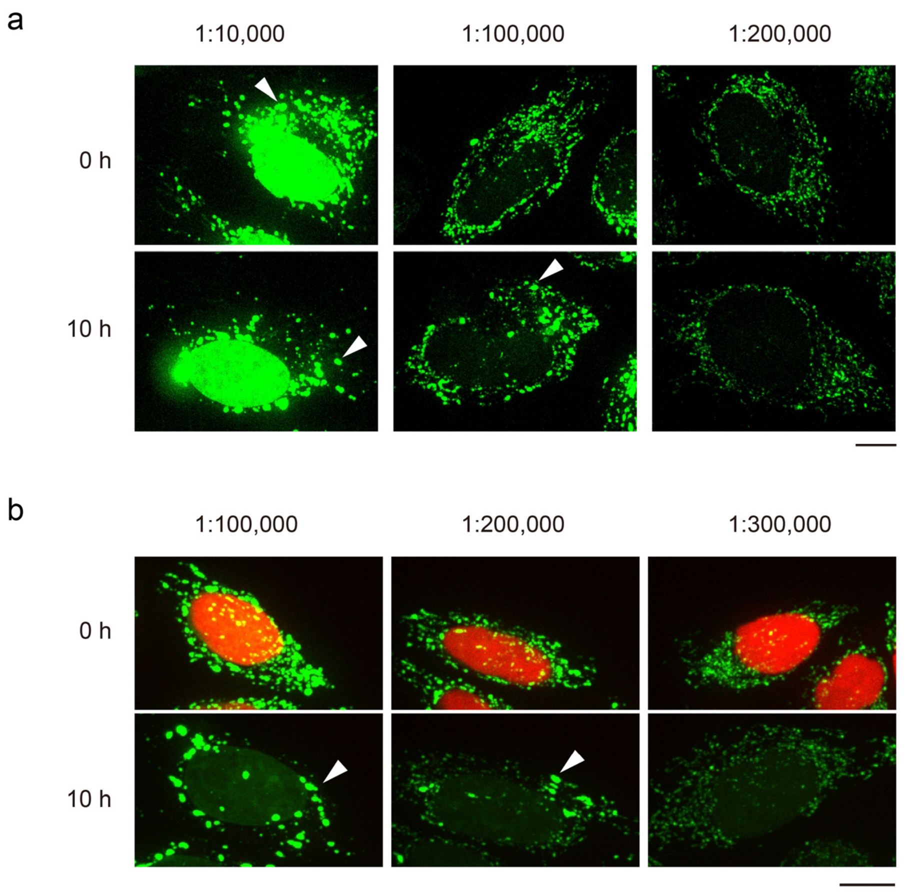

Figure S1| Staining pattern of SYBR Green I in Fucci2 cells. (a) Time-lapse imaging of SYBR Green I stained normal HeLa cells. Normal HeLa cells were stained with various concentration of SYBR Green I and subjected to time-lapse imaging at 10-h intervals. Dilutions of SYBR Green I are indicated above the images. Arrowheads show enlarged mt-nucleoids. Scale bar, 10 µm. **(b)** Time-lapse imaging of SYBR Green I stained Fucci2 cells. Fucci2 cells were stained with various concentrations of SYBR Green I and subjected to time-lapse imaging at 10-h intervals. The optimum concentration of SYBR Green I in Fucci2 cells was 1:300,000 dilution in Fucci2 cells. The concentration of SYBR Green I is shown in the upper part of the images. Arrowheads show enlarged mt-nucleoids. Scale bar, 10 µm.

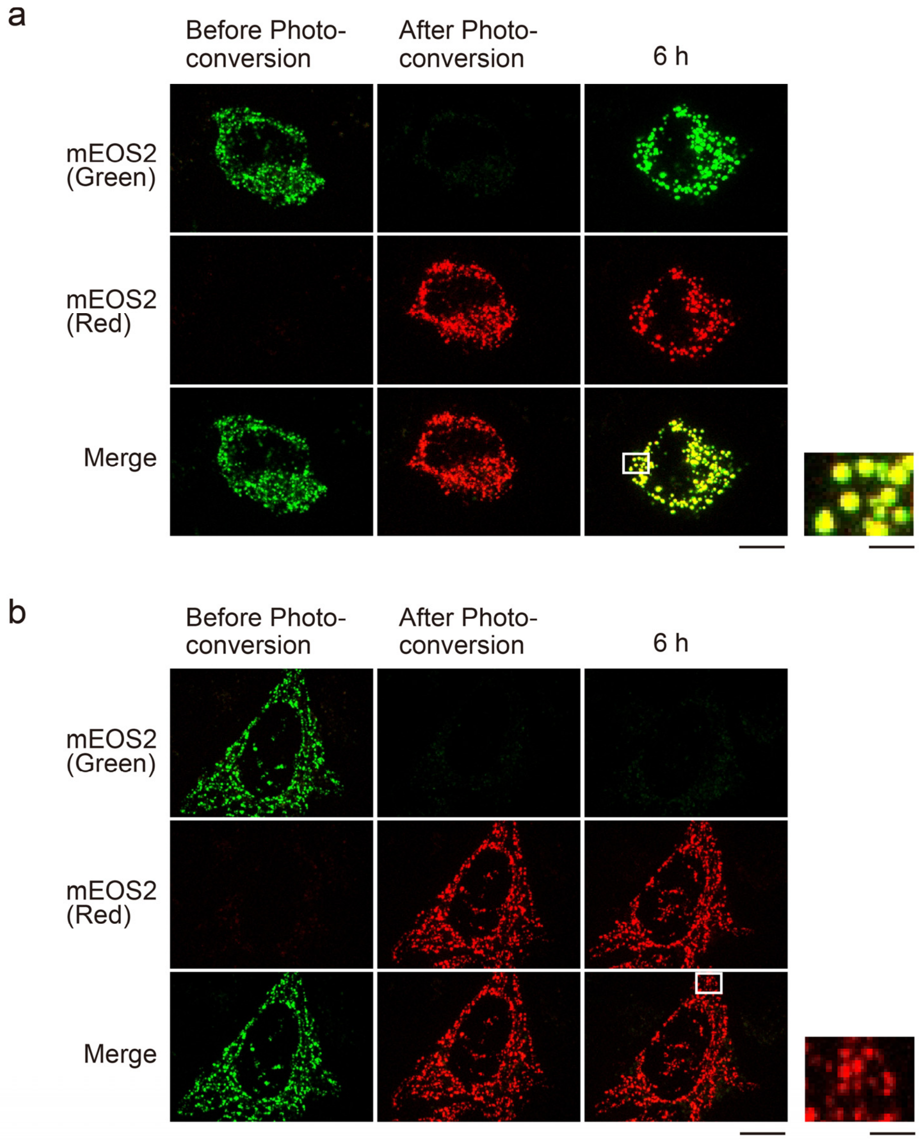

Figure S2| TFAM-mEOS2 expression was controlled by the GeneSwitch system. (a) Effect of continuous expression of TFAM-mEOS2. TFAM-mEOS2 cells were incubated with mifepristone to induce TFAM-mEOS2. Then, the entire cell was photoconverted and observed. The right panel is an enlarged view of the merged image of 6 h. Six hours after photoconversion, enlarged mt-nucleoids (arrowhead) and de novo TFAM-mEOS2 were observed. Scale bar, 10 μm for images of entire cells, and 2 μm for the enlarged view. **(b)** Effect of limited expression of TFAM-mEOS2. TFAM-mEOS2 cells were incubated with mifepristone and incubated in the absence of mifepristone for 5 h to stop this induction. Then, the entire cell was photoconverted and observed after 6 h. The right panel is an enlarged view of the merged image of 6 h. Scale bar, 10 μm for images of entire cells, and 2 μm for the enlarged view.

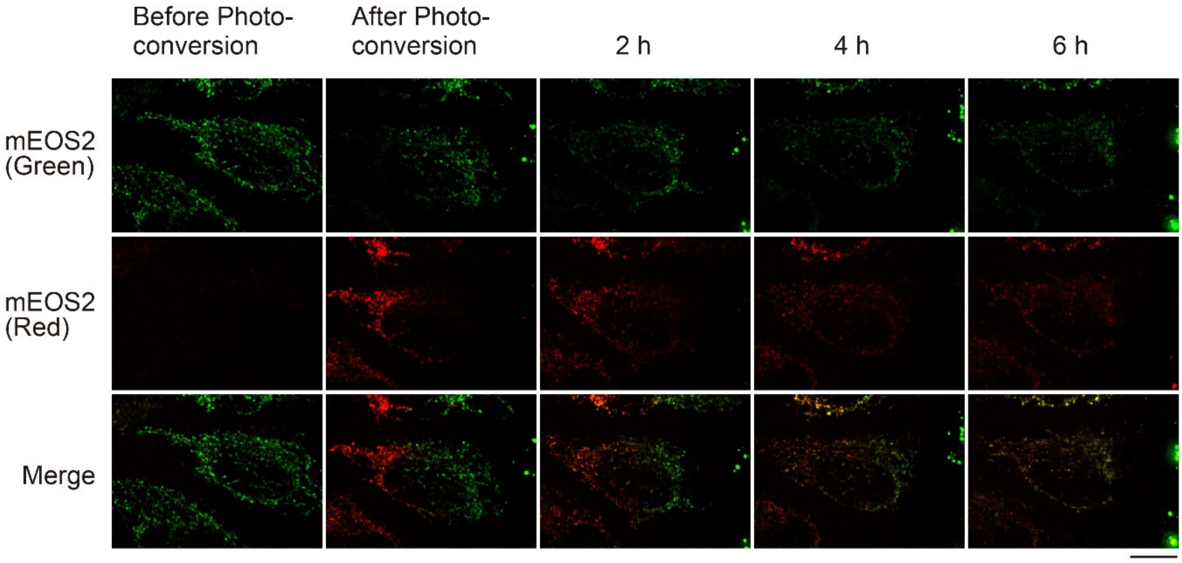

Figure S3| Green and red versions of TFAM-mEOS2 mixed during long-term observation. TFAM-mEOS2 cells were incubated with mifepristone to induce TFAM-mEOS2 expression, and incubated in the absence of mifepristone for 5 h to stop this induction. Half of the cells were photoconverted, and time-lapse imaging was performed at 2-h intervals. Scale bar, 10 μm.

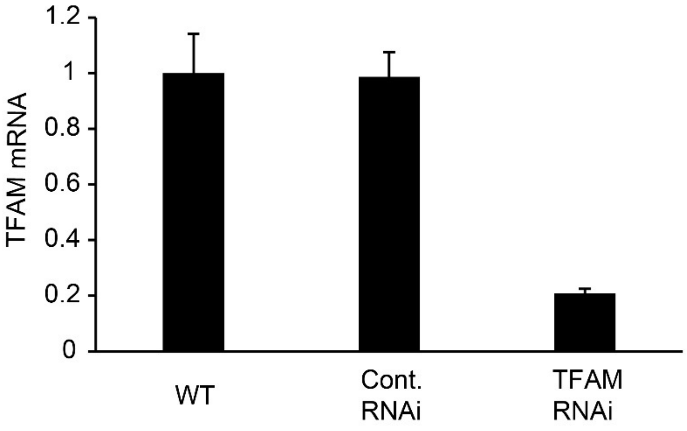

Figure S4| TFAM expression level was reduced by TFAM RNAi. Knockdown of TFAM was performed in HeLa cells. mRNA levels were quantified using RT-qPCR at 10 h after transfection of siRNAs. TFAM mRNA levels relative to those in wild-type cells are shown. Error bars indicate standard deviation (n = 3 independent experiments).

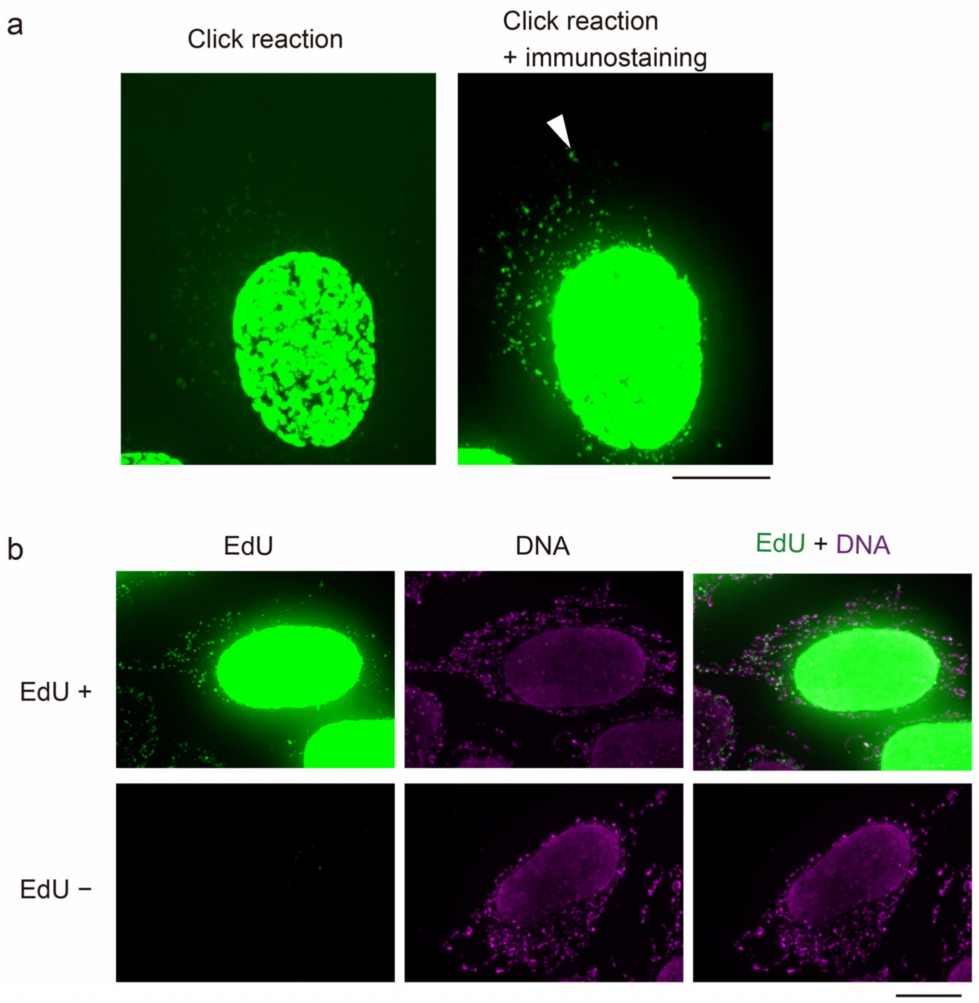

Figure S5| mtDNA replication was visualized using EdU in Fucci2 cells (a) Amplification of EdU signals by immunostaining. Fucci2 cells were incubated with 20 µM EdU for 1 h. After the click reaction, only faint signals were observed around the nucleus (left panel). Many bright spots in the cytosol (arrowheads) were observed in the same cell after signal amplification by immunostaining (right panel). Scale bar, 10 µm. **(b)** Double staining of EdU and DNA by immunofluorescence microscopy. Fucci2 cells were incubated with (EdU +) or without (EdU −) 20 µM EdU for 1 h. Then, we performed immunostaining of DNA using anti-DNA antibodies (magenta) and signal amplification of EdU (green). Scale bar, 10 µm.

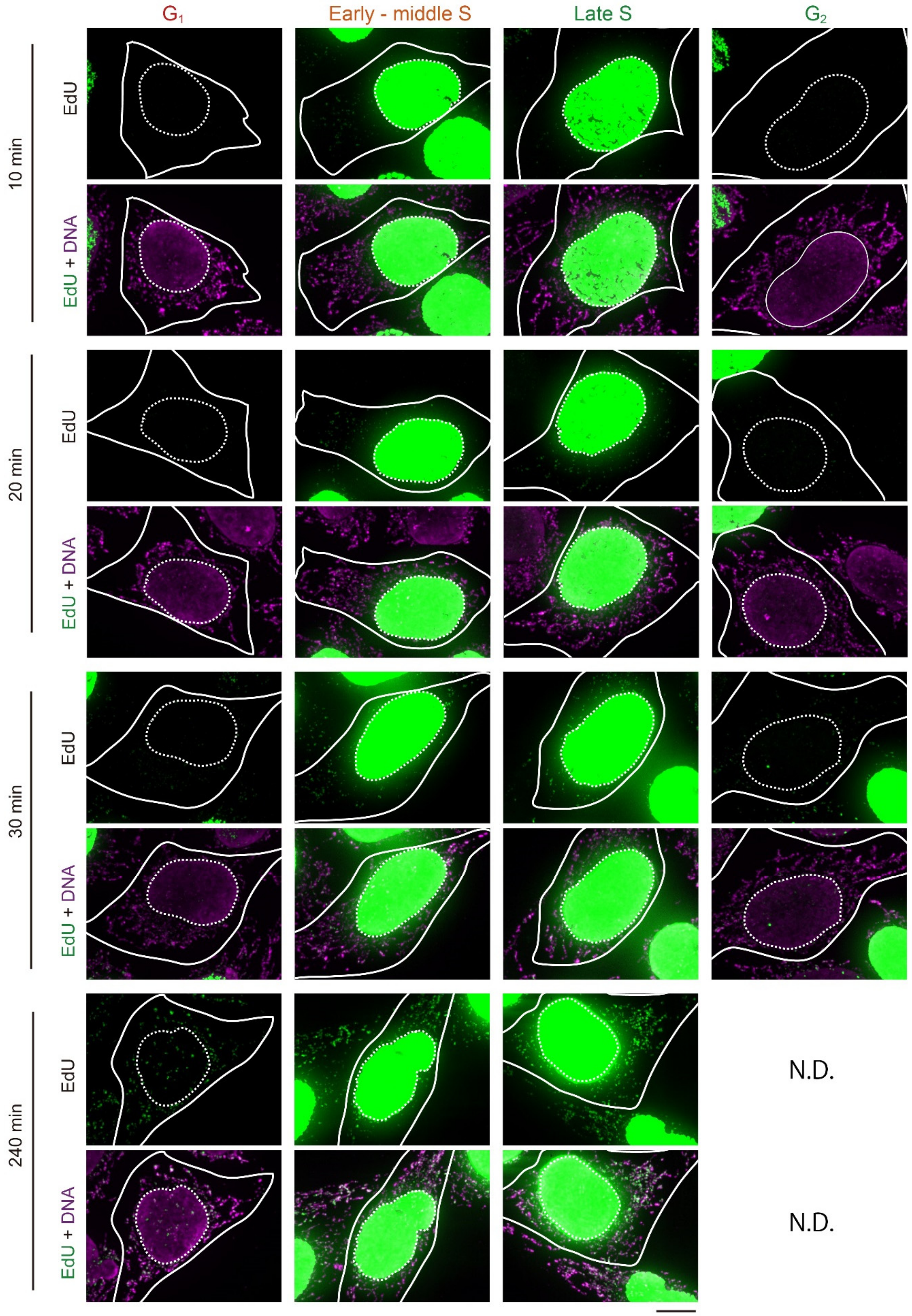

Figure S6| EdU incorporation in HeLa.S-Fucci2 cells. Fucci2 cells incubated with EdU for 10–240 min. Under the conditions with 240 min of incubation, we could not detect G_2-phase cells because the duration of the G_2 phase was shorter than 240 min. N.D.: not detected. Position of the nucleus (white dotted line) and cell shape (white line) are shown in each image. Scale bars, 10 μm.

Supplementary Video Legends

Video 1| Time-lapse imaging of mt-nucleoids and mitochondrion. Images correspond to the mitochondria (red) and mt-nucleoids (green) shown in Figure 2A. mtDNAs were stained with SYBR Green I in HeLa cells expressing mitochondrially targeted DsRed (HeLa-Su9 cells). Images were acquired using confocal microscope. Scale bar, 1 μm.

Video 2| Time-lapse imaging of TFAM-mEos2 cells. Images correspond to the mt-nucleoids shown in Figure 3A. Time-lapse imaging of mt-nucleoids in a TFAM-mEOS2 cells using confocal microscope. Partial region of the cell was irradiated by a laser at 405 nm for photoconversion. Change of the colors of mt-nucleoids was not observed after attachment. Scale bar, 1 μm.

Supplementary Method

RT-qPCR of TFAM-knockdown cells. For RT-qPCR, HeLa-Su9 cells were plated onto six-well plate (Corning, 1.25×10^5 cells per well) and cultured for 1 day. Next, siRNAs (final conc. 20 nM) were transfected as described in Methods section, and incubated for 10 h. Then total RNA was extracted from 1×10^5 cells using RNeasy mini kit (QIAGEN, Germany), and reverse transcription was performed using High-Capacity RNA-to-cDNA Kit (Thermo Fisher Scientific). RT-qPCR was performed using SYBR Premix Ex Taq II (Takara, Japan). Primer sequences were 3'- GCTCAGAACCCAGATGCAAA-5' and 3'- CTGCCACTCCGCCCTATAA-5' for *TFAM*, and 3'- AAACTGGAACGGTGAAGGTG-5' and 3'- GAGGCTTTTAGGATGGCAAG-5' for *ACTB*. Reactions were analyzed upon Step One Plus (Applied Biosystems, U.S.A.). Results were normalized against *ACTB*.
